# Translational Results of Zo-NAnTax: A Phase II Trial of Neoadjuvant Zoledronic Acid in HER2-Positive Breast Cancer

**DOI:** 10.3390/ijms232415515

**Published:** 2022-12-08

**Authors:** Susanne Crocamo, Renata Binato, Everton Cruz dos Santos, Bruno de Paula, Eliana Abdelhay

**Affiliations:** 1 Núcleo de Pesquisa Clínica, Hospital de Câncer III, Instituto Nacional de Câncer José Alencar Gomes da Silva, Rio de Janeiro 20560-121, Brazil; 2 Laboratório de Célula-Tronco, Instituto Nacional de Câncer José Alencar Gomes da Silva, Rio de Janeiro 20230-130, Brazil

**Keywords:** breast cancer, HER2-positive, differential expression genes, zoledronic acid

## Abstract

Breast cancer is a heterogeneous disease with distinct clinical and molecular characteristics. Scientific advances in molecular subtype differentiation support the understanding of cellular signaling, crosstalk, proliferation, survival, migration, and invasion mechanisms, allowing the development of new molecular drug targets. The breast cancer subtype with super expression and/or amplification of human growth factor receptor 2 (HER2) is clinically aggressive, but prognosis significantly shifted with the advent of anti-HER2 targeted therapy. Zoledronic-acid (ZOL) combined with a neoadjuvant Trastuzumab-containing chemotherapy regimen (Doxorubicin, Cyclophosphamide followed by Docetaxel, Trastuzumab) increased the pCR rate in a RH-positive/ HER2-positive subgroup, according to the phase II Zo-NAnTax trial. To verify genes that could be related to this response, a microarray assay was performed finding 164 differentially expressed genes. Silico analysis of these genes showed signaling pathways related to growth factors, apoptosis, invasion, and metabolism, as well as differentially expressed genes related to estrogen response. In addition, the RAC3 gene was found to interact with the MVD gene, a member of the mevalonate pathway. Taken together, these results indicate that RH-positive/ HER2-positive patients present gene alterations before treatment, and these could be related to the improvement of pCR.

## 1. Introduction

Breast cancer exhibits heterogeneous clinical behavior and treatment response, partially due to distinct molecular characteristics. Around 25% of breast cancer harbors amplification or super expression of human epidermal growth factor receptor 2 (HER2), a core driver for cellular growth, and about 50% of these cases concurrently express hormone receptors (HR). Regardless of hormonal receptor (HR) status, those tumors are associated with worse survival outcomes and intracranial recurrence [1,2,3]. 

Trastuzumab is a humanized monoclonal antibody that significantly improves outcomes of HER2-positive tumors either alone or in combination with chemotherapy [4,5]. Multiple antitumor mechanisms are attributed to Trastuzumab, including deregulation of HER2 expression, inducing internalization, and degradation of the receptor. By binding to domain IV of the HER2, it inhibits homodimerization and blocks pathways PI3K/Akt/mTOR and Ras/Raf/MAPK, also inhibiting angiogenesis, proliferation, and metastasis [6].

Despite the significant benefits induced by Trastuzumab, treatment failures are still frequent, either by reactivation of the HER2 pathway, activation of compensatory pathways, or redundancy of alternative survival pathways [7]. Therefore, subsequent drugs were developed such as Pertuzumab, another monoclonal antibody that binds in a different domain of HER2, and which is an added benefit when combined with Trastuzumab-containing regimens. The tyrosine kinase inhibitors (Lapatinib, Neratinib, and Tucatinib) and antibody-drug conjugates (Ado-Trastuzumab Emtansine, Fam-Trastuzumab, Deruxtecan-nxki, and Margetuximab-cmkb) [8] have also emerged as having a meaningful effect but with a significant increase in cost and in some cases life-threatening toxicity. 

Neoadjuvant studies are crucial to breast cancer research. They provide an opportunity to evaluate tumors sample before and after treatment and are an outstanding platform for identifying new biomarkers, predictors of response, mechanisms of drug resistance, developments of investigational drugs, triaging of novel combinations, and drug repositioning. This approach is mainly considered in-clinic for triple-negative and HER2-positive breast cancer subtypes, where pathological complete response (pCR) is a good surrogate of survival benefit [9].

Several neoadjuvant studies associating chemotherapy with single or dual HER2 blockade showed a significant increase in the pCR rate of all populations studied. However, when they were evaluated according to HR positivity, there was a significantly lower pCR rate for HR-positive tumors compared to HR-negatives, as can be seen in Table 1. These results demonstrate the need for a greater understanding of this tumor subtype. While there has been increased benefit with the new therapies, several unmet needs remain, such as the establishment of biomarkers that would allow the de-escalation of anti-HER2 treatment and/or chemotherapy [10,11], the determination of which other pathways could be concurrently or sequentially blocked to circumvent resistance, and the role of repurposing drugs.

The mevalonate biosynthetic pathway (MVA) regulates cholesterol production and participates in post-translational modifications of Rho-GTPases, which are isoprenylated metabolites, essential for tumor cell growth and progression. Inhibition of the MVA pathway can reduce the isoprenylation of these small GTPases and induce cell death [18]. However, the complexity of this pathway is notable, and how lipid metabolism relates to tumor development and progression, as well as the best way to target it, is currently under investigation [19].

Bisphosphonates inhibit mevalonate metabolism [20]. In pre-clinical studies, zoledronic acid (ZOL), a third-generation bisphosphonate, has inhibited tumor cell proliferation, induced apoptosis, inhibited angiogenesis, reduced cell invasion and migration, activated specific antitumor cellular immune responses, and induced synergistic/additive antitumor effect with anthracycline and paclitaxel [21].

In the phase II Zo-NAnTax trial [17], we assessed the benefit in pCR in HER2-positive breast cancer by adding zoledronic acid (ZOL) to a neoadjuvant treatment regimen based on anthracycline + cyclophosphamide followed docetaxel + trastuzumab. Unlike previous studies (Table 1), we observed a meaningful increase in the pCR rate in the RH-positive subgroup (40%), which reached levels comparable to the RH-negative subgroup (44%).

In this article, we report the molecular signatures of differentially expressed genes (DEGs) from HR-positive/HER2-positive tumors of patients who achieved pCR versus those who did not, investigating their gene-to-gene interactions. We investigated their involvement in biological processes and signaling pathways, aiming to understand the mechanism of associating ZOL in increasing pCR in HR and HER2-positive breast cancer. 

## 2. Results

### 2.1. Clinical Results

The Zo-NAnTax trial [17] achieved its primary endpoint by showing a pathological complete response (pCR) rate (42%) across the entire patient population. Secondarily, there was a similar pathological response rate in the hormone receptor (HR)-positive (40%) subgroup as compared to the negative HR (44%) subgroup, unlike the results in the literature. Here, we report the gene expression analysis of 16 patients randomly selected from the subgroup of RH-positive/HER2-positive patients from the Zo-NAnTax trial to identify molecular differences that could justify this improvement in pCR.

The baseline characteristics of these patients and their tumors are described in Table 2. Sixteen patients with estrogen-receptor (ER) and/or progesterone-receptor (PgR)-positive disease. The median age was 56.0 (26.0–74.0) years and the tumors were large, with a median size of 57 mm. A total of 10 patients (62.5%) were postmenopausal. Most patients had stage IIB (37.5%) cancer, tumor grade 2 (62.5%). A total of four patients (25%) had a family history of breast/ovarian cancer. TILs were present in 10 patients (62.5%). Most patients had KI67 ≥ 20% (84%) and 11 patients (64%) had p53 ≥ 10.

All 16 (100%) patients had a breast operation performed. pCR (RCB 0) was achieved in seven patients (44%). Fourteen patients (87.5%) were alive at five years and without disease recurrence (Table 3).

### 2.2. Microarray Analysis Revealed Differentially Expressed Genes at Diagnosis Related to Response

To verify if HR-positive/HER2-positive patients could present, before treatment, differentially expressed genes that could be related to the response, we performed the global gene expression pattern from HR-positive/HER2-positive patients that had pCR. We compared this with the global gene expression pattern from HR-positive/HER2-positive patients who achieved versus those who did not achieve pCR, using microarray assay. 

In the transcriptomic analysis, we used seven biopsy samples without any treatment from HR-positive/HER2-positive patients that had pCR and compared these with nine biopsy samples without any treatment from HR-positive/HER2-positive patients who did not achieve pCR. For this, the total RNA that was obtained from each previously frozen biopsy was processed and hybridized to Human Gene Expression v2 4 × 44K microarrays according to the manufacturer’s protocols. Using a ≥2-fold change and *p* < 0.05 as a cut-off to define overexpression or downregulation, 164 genes (Supplementary Table S1) were found to be differentially expressed in HR-positive/HER2-positive patients who achieved pCR in comparison with those who did not achieve this response. Among these 164 differentially expressed genes, 86 were upregulated and 78 were downregulated.

### 2.3. Differentially Expressed Genes Indicate Growth Factors and Metabolism Signaling as Those Pathways Related to Zo-NAnTax Response 

In order to identify signaling pathways that could be related to differentially expressed genes found in HR-positive/HER2-positive patients who achieve pCR in comparison with those who do not, we performed an in silico analysis using Webgestalt software, the “WEB-based GEne SeT AnaLysis Toolkit” (http://www.webgestalt.org/ (accessed on 24 August 2022)). WebGestalt is a free web-tool that helps to interpret high-throughput experiments through a gene set enrichment analysis tool that enables the use of different databases to extract different biological insights from a given gene list. When we used Panther, Wikipathway, and KEGG databases we found that different pathways were enriched depending on the database used, which is a very insightful way to visualize how our differentially expressed genes can interplay and impact relevant biological pathways. The Panther database showed signaling pathways related to growth factors such as VEGF and PDGF, signaling pathways involving important regulatory genes such as: SHC Adaptor Protein 2 (SHC2), a member of the Src homology and collagen (SHC) family, which are essential elements in signaling cascades; RAS-like Estrogen Regulated Growth Inhibitor (RERG); Rac family small GTPase 3 (RAC3), a GTPase which belongs to the RAS superfamily of small GTP-binding proteins. SHC2 presented upregulated and RAC3 downregulated. In this analysis we also identified P53 and ubiquitin proteasome pathways presenting differentially expressed genes: P53 targeted genes such as p53 apoptosis effector related to PMP-22 (PERP) and Cluster of differentiation 82 (CD82), a metastasis suppressor gene, presented upregulated. We also observed that, interestingly, Wikipathway and KEGG databases showed mainly pathways related to metabolism associated with the antioxidant response and detoxification of xenobiotics (NRF2 pathway, Arachidonate Epoxygenase/Epoxide Hydrolase, and metabolism of xenobiotics by cytochrome P450), which are intimately related to the other pathways identified relating to drug metabolism (Table 4 and Supplementary Figures S1–S6). We identified that some genes known for their function in detoxification activity and the metabolism of endogenous and exogenous toxic compounds connect those pathways, such as the downregulated genes Glutathione S-Transferase Pi 1 (GSTP1), Gamma-Glutamyltransferase Light Chain 1 (GGTLC1), the Carbonyl Reductase 3 (CBR3), and the upregulated genes, such as Cytochrome P450 Family 2 Subfamily B Member 6 (CYP2B6) and Glutathione S-Transferase Theta 1 (GSTT1). 

### 2.4. SLC9AR1 Is Highlighted as a Possible Candidate in HR-Positive/HER2-Positive Patient Outcome

**Figure 1:**
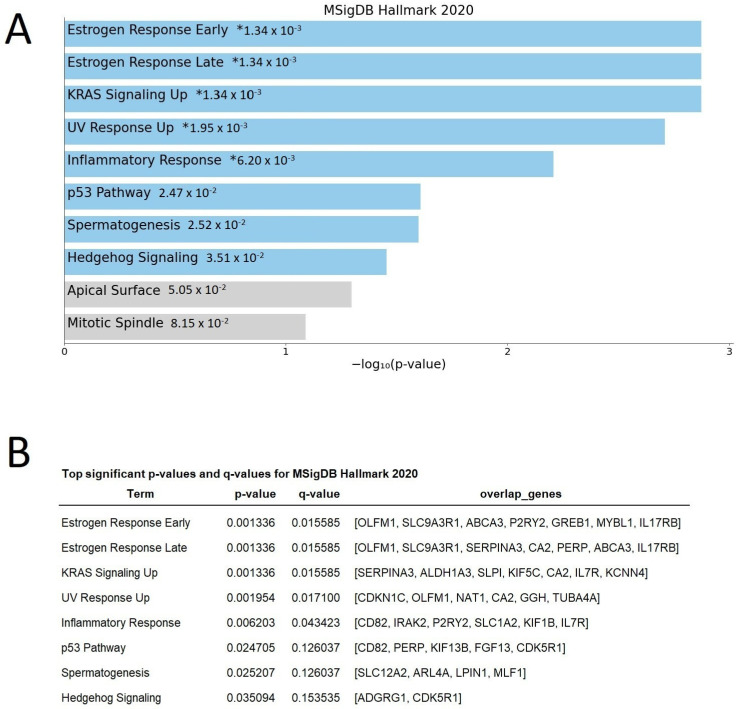
MSigDB Hallmark 2020 analysis. (**A**) Bar chart of top enriched terms from the MSigDB Hallmark 2020 gene set library. The top 10 enriched terms for the input gene set are displayed based on the −log10 (*p*-value), with the actual *p*-value shown next to each term. The term at the top has the most significant overlap with the input query gene set. The asterisk (*) indicates a significant adjusted *p*-value (<0.05). The adjusted *p*-value is computed using Benjamini-Hochberg method for correction for multiple hypothesis testing (**B**) Table displaying the names, *p*-values, and q-values of significant terms and the genes from the input that were found to be associated with that term. The q-value is an adjusted *p*-value calculated using the Benjamini-Hochberg method of correction for multiple hypothesis testing. Only the top significant results are displayed.

We also performed an in silico analysis using the website tool Enrichr to search terms in the Molecular Signatures Database gene-set library (MSigDB), a free database of collections of different gene sets, where we used the MsigDB Hallmark 2020 collection. This database collection comprises annotated gene sets representing specific, well-defined biological states or processes displaying coherent expression. These gene sets were generated by a computational methodology based on identifying overlaps between gene sets in other MSigDB collections and retaining genes that display coordinate expression. In this analysis, it was interesting to observe that two of the main processes related to response were “Estrogen Response Early” and “Estrogen Response Late” and most of the differentially expressed genes related to both estrogen responses showed decreased expression (13 of 14 genes) (Figure 1).

**Figure 2:**
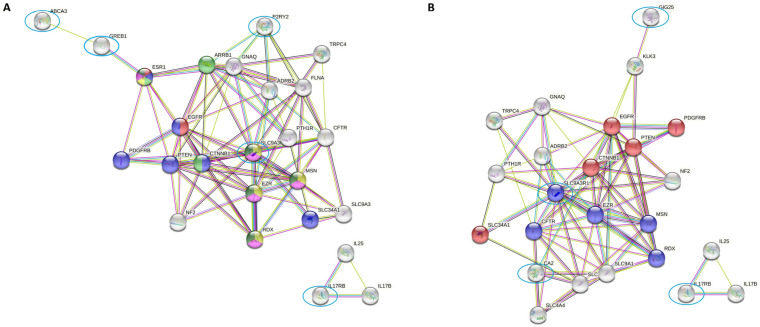
Network of interactions among the differentially expressed genes related to (**A**) “Estrogen Response Early” and (**B**) “Estrogen Response Late” by STRING software. The colored balls represent proteins related to the estrogen pathway. Downregulated genes are marked with blue circles. Associations were visualized with a medium confidence cutoff (0.400).

As these processes are relevant to the investigation of breast cancer from a targeting perspective, the 14 DEGs identified in these processes may be somehow related to estrogen response. Thus, we analyzed the proteins coded by those genes through a protein-protein interaction network in the STRING software. Since the relationship of those proteins with the estrogen response in our sample is unknown, we included in the analyses our 14 query proteins, and we enabled the first shell of interactors with a maximum of 20 non-query proteins (from the software database) to find the main proteins related to our protein set. As shown in Figure 2, we divided the genes presented in “Estrogen Response Early” and “Estrogen Response Late” in two STRING analyses using strong interaction data (curated databases, experimental determination, gene neighborhood, gene fusions, text mining, co-expression, protein homology, gene co-occurrence) in the software as evaluation criteria. All colored genes are directly or indirectly related to the estrogen pathway. Intriguingly, the SLC9A3R1 protein was downregulated in HR-positive/HER2-positive patients who achieved pCR, shown as a key hub in both STRING analyses, suggesting a role of this gene in inducing treatment resistance in this subset of tumors. 

**Figure 3:**
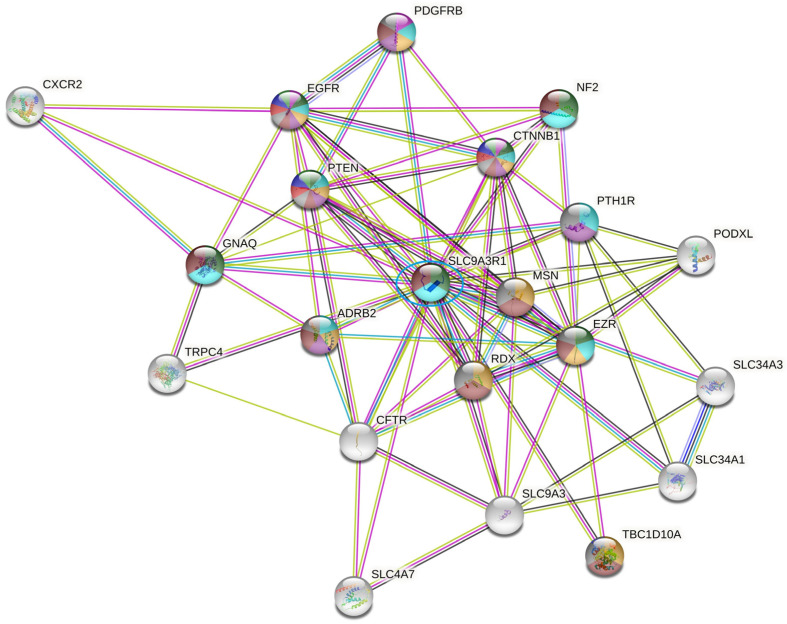
Network of interactions among SLC9A3R1 proteins by STRING software. The colored balls represent proteins related to the estrogen pathway. Associations were visualized with a medium confidence cutoff (0.400).

To identify the proteins that interact with SLC9A3R1 we performed a STRING analysis using the same criteria that we used before. As seen in Figure 3, SLC9A3R1 interacts with several proteins related to the estrogen pathway and tumorigenesis, including EGFR, PTEN, PDGFR, B-catenin, and ezrin.

### 2.5. RAC3 Gene Links Growth Factor Signaling with Mevalonate Pathway

**Figure 4:**
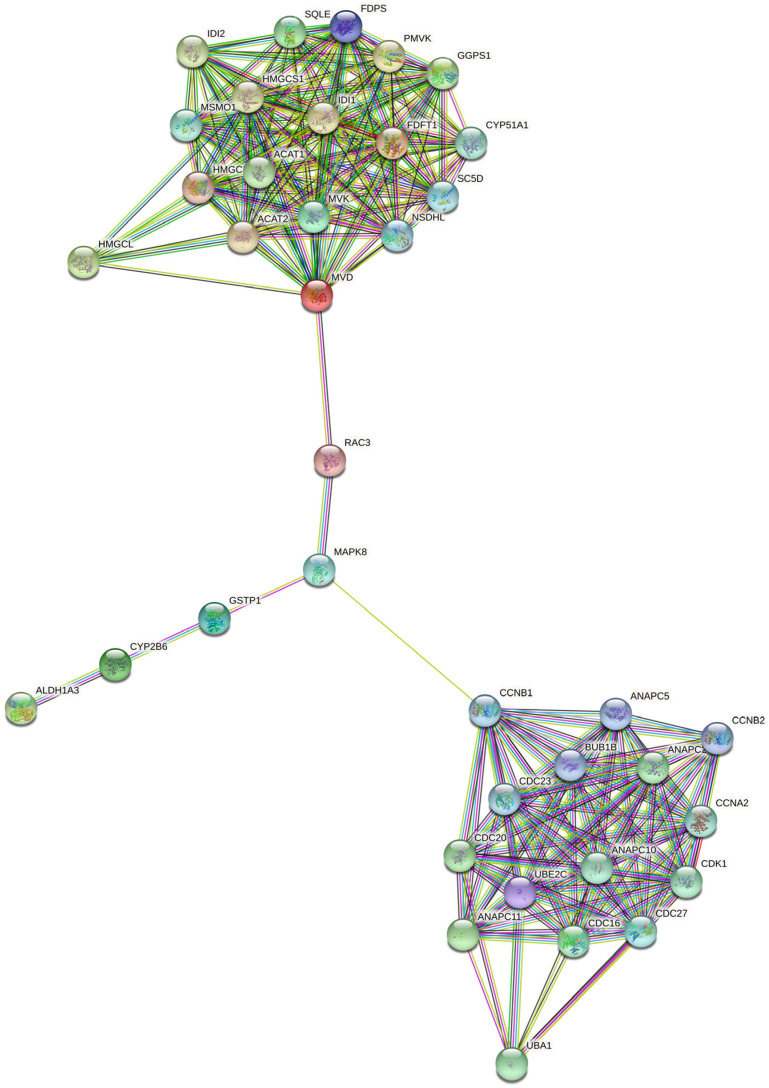
Network of interactions among mevalonate pathways and differentially expressed genes presented in the pathways obtained in Webgestalt by STRING software. Associations were visualized with a medium confidence cutoff (0.400).

The Zo-NAnTax clinical trial evaluated the association of zoledronic acid (ZOL), chemotherapy, and HER2 target therapy [17]. As is already known, ZOL blocks the mevalonate pathway and could interact with the HER2 pathway. Therefore, it would be interesting to assess whether there is any relation among the differentially expressed genes found in HR-positive/HER2-positive patients that obtained complete responses with the mevalonate pathway. For this, we selected the differentially expressed genes in the pathways obtained in Webgestalt and put them together with mevalonate-related genes in a STRING analysis. As can be seen in Figure 4, the RAC3 gene (decreased in HR-positive/HER2-positive patients who achieved pCR) interacts with the MVD gene from the mevalonate pathway.

## 3. Discussion

The Zo-NAnTax was the first clinical trial to prospectively assess the in vivo benefit of ZOL in a neoadjuvant treatment setting combined with chemotherapy and HER2-targeted therapy for HER2-positive breast cancer. This study achieved its primary objective of therapeutic efficacy, with an overall pCR rate of 42%. However, different from the literature studies, our results showed that pCR rates according to HR status were similar between HR-positive and negative subgroups (40% versus 44%, respectively) [17].

Around 60% of HER2 positive breast cancer also expresses hormonal receptor (HR) positivity. When both ER and HER2 pathways are activated, there is a bi-directional cross-talk where blocking one might induce super activation of the other [22].

The estrogen pathway mediates biological effects, including signal transcription and transduction, by ER-dependent or -independent stimulus. When a ligand binds to an ER, a cascade of transcription gene activation is initiated, resulting in proliferation and survival, inhibition of anti-proliferation, or pro-apoptosis. Beyond that, it can regulate gene expression without directly binding to the DNA, modulating the function of other transcriptional factor classes by interactions among proteins at the cellular nucleus [23].

Considering the bi-directional crosstalk between HER2 and ER pathways, the signaling by different growth factor receptor-dependent kinases downstream phosphorylates several ER cofactors, including the ER itself [24].

This potentiates ER genomic signaling activity in gene transcription independent of the presence of ligand or selective ER modulators. In consequence, this can directly or indirectly activate the epidermal growth factor receptor (EGFR), HER2, and insulin-like growth factor receptor 1 (IGFR1), leading to a cascade of activations downstream of the RAS/MEK/protein pathway mitogen-activated kinases (MAPK) and PI3K/AKT/mTOR [25].

Wang et al. [26] showed that resistant cells reactivated the HER pathway as a resistance mechanism after trastuzumab treatment; however, dual-blockade (Lapatinib and Trastuzumab) resistance required the activation of an alternative pathway of escape. As seen in four of the five ER-positive/HER2-positive cell lines, the ER and its downstream products increased, demonstrating a reactivation of ER expression and signaling.

The survival benefit gained in achieving pCR for triple-negative and HER2-positive tumors is unquestionable, especially in the HER2-enriched subgroup [9]. However, for the HR-positive/HER2-positive subgroup, the magnitude of this benefit has often been questioned. 

Since pCR also plays an essential role in a long-term better outcome in the HR-positive/HER2-positive subgroup of BC patients [27,28], we explored the benefits of pCR improvement in the HR-positive/HER2-positive subgroup of the Zo-NAnTax trial. Therefore, we performed a microarray assay to verify if there would be differentially expressed genes between patients who achieved pCR and those who did not that could predict this increase in pathological response. 

We found 164 differentially expressed genes (DEGs). In silico analysis of these genes revealed interesting pathways in which these differentially expressed genes could be related. DEGs were significantly enriched in biological processes such as “Estrogen Response Early”, “Estrogen Response Late”, KRAS signaling up, inflammatory response, and the p53 pathway. They are crucial processes for breast oncogenesis. In addition, studies in RH-positive/HER2-negative cells have shown that “Estrogen Response Early” can provide a score of genes involved in a better-targeted therapeutic response and survival gain [29]. Interestingly, we can observe that several genes are common to different biological processes. 

Differently expressed genes of the main processes relating to either “Estrogen Response Early” or “Estrogen Response Late” were found (Figure 1). Surprisingly, from the total of 14 genes of both estrogen responses, we found 13 genes with a reduced response. Of the 7 genes differentially expressed in “Estrogen Response Early”, it is important to highlight the reduction in GREB1 expression. In ER-positive breast cancers, the plasma level of estradiol and the expression of ERs are positively correlated with GREB1 expression [30,31]. An optimal level of GREB1 expression is necessary for the proliferation of breast cancer cells through PI3K/Akt/mTOR pathway signaling. However, the molecular function by which GREB1 regulates proliferation is unknown.

Experiments performed in GREB1 knockdown MCF-7 cells showed that almost half of the estrogen responsive genes were no longer differentially expressed, and the cells were less able to form colonies [31]. This would be a mechanism that could have helped our patients to achieve pCR since in our samples it was found to be downregulated. Nevertheless, the involvement of GREB1 is much more complex. A more recent study showed that when knocking down GREB1, the proliferation of estrogen-dependent breast cancer cells is rescued by the expression of Akt constitutively across the direct link between estrogen signaling and the PI3K pathway [32]. 

At that time, if this escape pathway is activated, we can assume that the benefit of zoledronic acid involves the suppression of MEK inhibitor-induced Akt activation, leading to combined apoptosis dependent on the inhibition of geranylgeranylation [33,34].

Regarding differentially expressed genes in “Estrogen Response Late”, the increase in PERP expression caught our attention. PERP is a component of desmosomes, multiprotein complexes involved in cell-to-cell adhesion. It is upregulated in p53-dependent cell death and during E2F1-induced cell death. In cells with HER2, overexpression is involved in the anoikis in a pathway by unknown mechanisms [35].

The PERP overexpression evidenced in our results favors an improvement in the sensitivity of transformed cells to inhibitors of mevalonate’s pathway due to the inhibition of downstream metabolic products. This can induce apoptosis, increasing intracellular ROS generation and p38 activation and suppressing the activation of Akt and Erk pathways [36].

In our results, the interleukin receptor 7 (IL7R) gene was upregulated and was part of two groups of biological processes: “KRAS signaling up” and “Inflammatory Response”. Its signals through the JAK/STAT pathway after dimerization induce the formation of cholesterol-enriched membrane microdomains (lipid rafts) and the approximation and reciprocal activation of JAK1 and JAK3, followed by the phosphorylation of IL7Rα’s residue, Y449. Y449 phosphorylation also recruits and activates the PI3K/AKT pathway, which could lead to increased therapeutic resistance [37].

However, our patients had an excellent therapeutic response, which leads us to suppose that there could have been a proliferation blockage by an alternative pathway. The mechanism of RAS activation by IL7R is unknown, but it is suggested that tyrosine phosphorylation on IL7R, JAK1, or JAK3 provides binding sites for adapter proteins such as Shc and/or Grb2, which upon phosphorylation recruit SOS to the plasma membrane; this in turn activates RAS. Thus, the inhibition of RAS prenylation would block its activation and could help to inhibit cell proliferation of the transformed cell and induce its death, adding to the benefits of pCR gain [37].

From the 14 DEGs identified in these processes, we analyzed the proteins encoded by them that could predict protein-protein interactions by STRING. The results showed that the SLC9A3R1 protein, downregulated in HR-positive/HER2-positive tumors in patients who achieved pCR, acts as a key hub in both STRING analyses.

SLC9A3R1 is a protein that joins plasma membrane proteins with members of the ezrin/moesin/radixin family and thereby helps to link them to the actin cytoskeleton and to regulate their surface expression. It is necessary for cAMP-mediated phosphorylation, the inhibition of SLC9A3, and could enhance WNT signaling. 

SLC9A3R1 protein expression was significantly related to an increase in undifferentiated tumor cells and to poor prognosis. Lower NHERF1 levels were identified only in ER-negative breast cancer lines. [38]. Cytoplasmic SLC9A3R1 was significantly associated with negative progesterone receptor (PgR) tumors and with HER2 overexpression, while nuclear SLC9A3R1 was associated with small -sized and positive ER tumors [39].

Studies in MDA-MB-231 cells demonstrated that the interaction of SLC9A3R1 with PTEN and BEC1 stimulates autophagy through the PTEN-PI3K-Akt signaling cascade [40]. Interestingly, in a pre-clinical study from Jaekwang et al. 2019 [41], the authors showed that treatment with an ezrin inhibitor plus lapatinib induced apoptosis of HER2-positive cancer cells. Considering this pre-clinical evidence, our translational data support the crucial role of SLC9A3R1 in HER2 stability and signaling. Once in HR-positive/HER2-positive patients where the SLC9A3R1 gene was downregulated, the combination of trastuzumab, chemotherapy, and zoledronic-acid—the latter affecting the mevalonate pathway—induced higher rates of pCR.

More specifically, we evaluated the network interactions between mevalonate pathways and differentially expressed genes present in the pathways obtained in Webgestalt using the STRING software (Figure 4). Interestingly, we found reduced RAC3 gene expression in the tumors of our HR-positive/HER2-positive breast cancer patients who achieved pCR, interacting with the MVD gene of the mevalonate pathway. Rac3, a member of the p21 Rho family of small GTPases, is an understudied paralog of the canonical Rac1 GTPase and has been implicated in cancer cell proliferation, invasion, and autophagy [20,42]. Few studies have assessed the role of Rac3 in breast cancer [42].

The PI3K–AKT signaling pathway is an important regulatory pathway for cell metabolism, protein synthesis, transcription, proliferation, and survival. In the cascade of phosphorylation events, the components of this pathway integrate, among others, signals from cell membrane receptors and receptor tyrosine kinases (RTKs) in response to growth factors [43].

Activating mutations in the PI3K-AKT pathway, inactivating the PI3K-AKT PTEN negative regulator, and/or hyperactivity of GFR-tyrosine kinases, in addition to the direct and independent binding of the p85 adapter of the small GTPase RAS to the subunit p110 catalytic activity of PI3K, are key mechanisms of PI3K activation [44].

Although compelling, the results of this study should be interpreted considering its limitations. Despite the samples being derived from patients enrolled in a prospective controlled phase II clinical trial with patient background, clinical characteristics, and therapeutic intervention data, the number of patients studied was small and it was not possible to evaluate the molecular results against the clinical variables. Nevertheless, this study was innovative in evaluating the DEGs of HR-positive/HER2-positive breast cancer patients who achieved versus those who did not achieve pCR after neoadjuvant treatment. 

Finally, our results demonstrated that mevalonate pathway blockage is important for treatment response in HR-positive/HER2-positive breast cancer. The mevalonate pathway can interact in several ways with the PI3k/Akt pathway, which is essential for both hormonal resistance and HER2 blockade resistance. It solidifies ZOL as a drug repositioned in breast cancer, with a positive impact on treatment benefits at lower clinical toxicity and financial cost.

## 4. Materials and Methods

### 4.1. Zo-NAnTAx Study Design

Seventy-one patients with HER2-positive BC stage IIA–IIIB signed the informed consent form (ICF) after the ethics committee and relevant health authorities approved the study and then were enrolled in the Zo-NAnTax neoadjuvant phase II trial, from November 2012 to July 2016. A total of 58 were eligible for the efficacy analysis. The design and results of the Zo-NAnTax trial were previously reported in detail [21]. In short, all 58 patients (42 RH-positive and 16 RH-negative) with HER2-positive locally advanced BC received four 3-week cycles of 60 mg/m^2^ doxorubicin and 600 mg/m^2^ cyclophosphamide (AC), followed by four 3-week cycles of 100 mg/m^2^ docetaxel (DOC) with 6 mg/ kg trastuzumab every 3 weeks (8 mg/kg as a loading dose). Overall, eight cycles of 4 mg/dose ZOL were given, the first cycle concomitant with AC and subsequent cycles occuring 1 week after each chemotherapy cycle (AC × 4 + ZOL × 4→DOC × 4 + trastuzumab × 4 + ZOL × 4) every 3 weeks; this was followed by surgery.

After surgery, radiotherapy was delivered according to institutional guidelines and trastuzumab was continued as a single agent for 1 year or combined with adjuvant endocrine therapy when indicated. Tissue samples were collected before and after treatment for histopathological diagnosis, immunohistochemistry, therapeutic response assessment, and molecular analysis.

We would like to highlight that neoadjuvant treatment with Trastuzumab and Pertuzumab together with chemotherapy had not yet been incorporated into the guidelines of our country during the conduction of this study.

### 4.2. Tissue Samples 

Core biopsies from the tumor and nontumoral areas were performed by guided ultrasonography following patient enrollment in the study. The decision between breast-conserving surgery and mastectomy or sentinel lymph node biopsy and axillary lymph node dissection was decided according to institutional guidelines. For both tissue samples, one part was paraffin-embedded for histological and immunohistochemistry (IHC) analysis, and the rest was frozen and stored at −80 °C. Histopathology and IHC analyses were performed pre- (core biopsy) and post-chemotherapy (surgical part material) by two experienced and blinded BC pathologists from our institution. The definition of pCR was an absence of infiltrating carcinoma in the breast and axilla.

### 4.3. Microarray 

The total RNA from the core biopsy before any treatment was extracted using an RNeasy Mini Kit (QIAGEN, Hilden, Germany). RNAs were quantified using a NanoDrop 2000 and RNA integrity was evaluated on a 2100 Bioanalyzer (Agilent Technologies, Santa Clara, CA, USA). A RIN higher than 6.0 was considered of enough quality to perform the microarrays. Biopsy RNA and a Universal Human Reference RNA (Stratagene, San Diego CA, USA) were amplified and differentially labeled with Cy5 and Cy3, respectively, using an Agilent Low Input Quick Amp Labeling Kit 2-Color, and subsequently hybridized 1:1 in mass (i.e., 825 ng each labeled RNA) with Human Gene Expression v2 4 × 44K microarrays, using the Agilent Gene Expression Hybridization Kit and Wash Buffer (Agilent Technologies, Santa Clara CA, USA). Feature Extraction 11.5.1.1 software (Agilent Technologies, Santa Clara, CA, USA) was used to generate raw data. GeneSpring software was used to analyze and identify the differentially expressed genes and ≥2-fold change and *p* < 0.05 were used as criteria to define overexpression or downregulation.

### 4.4. In Silico Analysis

The differentially expressed genes (DEGs) functional enrichment analysis was performed in the WEB-based Gene SeT Analysis Toolkit (WebGestalt, http://www.webgestalt.org/ (accessed on 24 August 2022)), a free online software tool that gathers information from various public databases for biological analysis [45]. We also performed Enrichment analysis using the integrative and collaborative website tool Enrichr (https://maayanlab.cloud/Enrichr/ (accessed on 24 August 2022)) using the pathways search module and Human Molecular Signatures Database gene-set library (MSigDB_Hallmark_2020) gene set library [46,47,48]. Enrichr results are corrected for multiple hypotheses using the Benjamini-Hochberg (BH) correction. Top terms with higher adjusted p values were chosen for subsequent interpretations. The protein-protein interaction (PPI) analyses of the proteins encoded by DEGs were predicted using Retrieval of Interacting Genes/Proteins (STRING) version 11.5 [49] against the human database. Associations were visualized with a medium confidence cutoff (0.400) using query proteins and the first shell of interactors with no more than 20 interactors. 

## 5. Conclusions

We found several DEGs between HR-positive/HER2-positive breast cancer patients who achieved versus those who did not achieve pCR and identified key hub genes, their associated biological processes, and the signaling pathways provided by a series of bioinformatic analyses. However, further molecular and biological experiments are required to confirm the pCR predictive value of these genes against treatment with the association of zoledronic acid to standard neoadjuvant treatment and for the assembly of a possible pathological response gene score.

## Figures and Tables

**Table 1:** Pathological complete response rates in HER2-positive breast cancer clinical trials according to hormone receptor expression.

Trials	Neoadjuvant Regimen	Overall	HR-Positive pCR Rate (%)	HR-Negative pCR Rate (%)
		pCR Rate (%)		
NEOSPHERE [12] Randomized/phase II	DocTP	39.3 ^&^	26.0 ^Ϯ^	63.2 ^Ϯ^
	DocT	21.5 ^&^	20.0 ^Ϯ^	36.8 ^Ϯ^
	TP	11.2 ^&^	5.9 ^Ϯ^	27.3 ^Ϯ^
	DocP	17.7 ^&^	17.4 ^Ϯ^	30.0 ^Ϯ^
NEOALTTO [13]	Pac * LT	46.8 ^&^	41.6 ^Ϯ^	61.3 ^Ϯ^
Randomized/	Pac * T	27.6 ^&^	22.7 ^Ϯ^	36.5 ^Ϯ^
phase III	Pac * L	20.0 ^&^	16.1 ^Ϯ^	33.7 ^Ϯ^
TRYPHAENA [14]	FECTP→DocTP	61.6 ^Ϯ^	41.1 ^Ϯ^	73.5 ^Ϯ^
Randomized/	FEC→DocTP	57.3 ^Ϯ^	45.7 ^Ϯ^	62.5 ^Ϯ^
phase II	DocCarbTP	66.2 ^Ϯ^	47.5 ^Ϯ^	81.1 ^Ϯ^
CALGB 40601 [15]	Pac * LT	52.0 ^&^	41.0 ^&^	68.0 ^&^
Ranzomized/	Pac * T	44.0 ^&^	39.0 ^&^	50.0 ^&^
phase III	Pac * L	27.0 ^&^	26.0 ^&^	30.0 ^&^
TECHNOS [16]	EC→Pac ** T	38.7 ^&^	35.4 ^&^	42.3 ^&^
Single arm/				
phase II				

Carb, carboplatin; Doc, docetaxel; EC, epirubicin/cyclophosphamide; FEC, fluorouracil/epirubicin/cyclophosphamide; L, lapatinib; P, pertuzumab; Pac, paclitaxel; pCR, pathological complete response; Trastuzumab; * Weekly paclitaxel; ** Paclitaxel every 3 weeks; ^Ϯ^ pCR only in breast (ypT0/ypTis); ^&^ pCR in breast and axilla (ypT0/ypTis ypN0). Adapted from [17]: *Ther. Adv. Med. Oncol.* **2019**, *11*, 1758835919853971.

**Table 2:** Patient and tumor characteristics at baseline.

Characteristics	HR-Positive
N (%)	(n = 16)
Age, mean (range), y	56 (26–74)
Menopausal Status, N (%)	
Premenopausal	6 (37.5)
Postmenopausal	10 (62.5)
Family History of Cancer, N (%)	
Breast/ovarian cancer	4 (25)
Any other type of cancer	6 (37.5)
No family history	6 (37.5)
T size, (range), mm	57 (30–90)
AJCC stage *, N (%)	
IIA	4 (25)
IIB	6 (37.5)
IIIA	2 (12.5)
IIIB	4 (25)
Histology, N, (%)	
Invasive ductal	16 (100)
Histologic grade ^†^, N (%)	
2	10 (62.5)
3	6 (37.5)
HER2, N (%)	
HER2 3 + (IHQ)	12 (75)
HER2 2 + (FISH positive)	4 (25)
TILs, N (%)	
Presence	10 (62.5)
Absence	6 (37.5)
Ki67 ^§^, N (%)	
<20	1 (6)
≥20	15 (84)
p53 ^§^, N (%)	
<10	5 (31)
≥10	11 (69)
Not performed	0 (0)

HR, hormone receptor; FISH, fluorescence in situ hybridization; HER2, human epidermal growth factor receptor 2; IHC, immunohistochemistry; TILs, Tumor-infiltrating lymphocytes; T, tumor. * TNM classification according to the International Union Against Cancer. ^†^ Grading according to Bloom–Richardson. ^§^ Immunohistochemistry was performed according to international guidelines.

**Table 3:** Patient outcomes after neoadjuvant treatment.

Characteristics	HR-Positive
N (%)	(n = 16)
RCB, N (%)	
RCB 0	7 (44)
RCB I	2 (12.5)
RCB II	5 (31)
RCB III	2 (12.5)
Alive at 5 years, N (%)	
Yes	14 (87.5)
No	2 (12.5)

RCB, Residual Cancer Burden.

**Table 4:** Signaling pathways related to the genes differentially expressed associated with response.

Signaling Pathways	Databank	Overexpressed Genes	Downregulated Genes
VEGF signaling pathway	Panther	SHC2	RAC3
p53 pathway	Panther	CD82-PERP	
PDGF pathway	Panther	SHC2	RERG
Ubiquitin proteasome pathway	Panther		UBE2C
Arachidonate Epoxygenase/Epoxide Hydrolase	Wikipathway	GSTP1	
Gamma-Glutamyl cycle for the biosynthesis and degradation of glutathione, including diseases	Wikipathway	GGTLC1	
NRF2 pathway	Wikipathway	CBR3-FGF13-GGTLC1-GSTP1-SLC2A9	
Nuclear receptors Meta-Pathway	Wikipathway	CBR3-FGF13-GGTLC1-GSTP1-SLC2A9-TNS4	CYP2B6
Metabolism of xenobiotics by cytochrome P450	KEGG	ALDH1A3-CBR3-GSTP1	CYP2B6-GSTT1
Drug metabolism	KEGG	ALDH1A3-GSTP1	CYP2B6-GSTT1
Drug metabolism 1	KEGG	GSTP1	GSTT1-NAT1

## Data Availability

The data presented in this study are available on request from the corresponding author. The data are not publicly available as they are a part of a work that has not yet been published.

## Supplementary Materials

The following supporting information can be downloaded at: https://www.mdpi.com/article/10.3390/ijms232415515/s1.

## References

[1] Slamon D.J., Clark G.M., Wong S.G., Levin W.J., Ullrich A., McGuire W.L. (1987). Human breast cancer: Correlation of relapse and survival with amplification of the HER-2/neu oncogene. Science.

[2] Slamon D.J., Godolphin W., Jones L.A., Holt J.A., Wong S.G., Keith D.E., Levin W.J., Stuart S.G., Udove J., Ullrich A. (1989). Studies of the HER-2/neu proto-oncogene in human breast and ovarian cancer. Science.

[3] Cheang M.C., Chia S.K., Voduc D., Gao D., Leung S., Snider J., Watson M., Davies S., Bernard P.S., Parker J.S. (2009). Ki67 index, HER2 status, and prognosis of patients with luminal B breast cancer. J. Natl. Cancer Inst..

[4] Gianni L., Eiermann W., Semiglazov V., Lluch A., Tjulandin S., Zambetti M., Moliterni A., Vazquez F., Byakhov M.J., Lichinitser M. (2014). Neoadjuvant and adjuvant trastuzumab in patients with HER2-positive locally advanced breast cancer (NOAH): Follow-up of a randomised controlled superiority trial with a parallel HER2-negative cohort. Lancet Oncol..

[5] Perez E.A., Romond E.H., Suman V.J., Jeong J.H., Sledge G., Geyer C.E., Martino S., Rastogi P., Gralow J., Swain S.M. (2014). Trastuzumab plus adjuvant chemotherapy for human epidermal growth factor receptor 2-positive breast cancer: Planned joint analysis of overall survival from NSABP B-31 and NCCTG N9831. J. Clin. Oncol..

[6] Hudis C.A. (2007). Trastuzumab—mechanism of action and use in clinical practice. N. Engl. J. Med..

[7] Rexer B.N., Arteaga C.L. (2012). Intrinsic and acquired resistance to HER2-targeted therapies in HER2 gene-amplified breast cancer: Mechanisms and clinical implications. Crit. Rev. Oncog..

[8] Wynn C.S., Tang S.C. (2022). Anti-HER2 therapy in metastatic breast cancer: Many choices and future directions. Cancer Metastasis Rev..

[9] Broglio K.R., Quintana M., Foster M., Olinger M., McGlothlin A., Berry S.M., Boileau J.F., Brezden-Masley C., Chia S., Dent S. (2016). Association of Pathologic Complete Response to Neoadjuvant Therapy in HER2-Positive Breast Cancer with Long-Term Outcomes: A Meta-Analysis. JAMA Oncol..

[10] Rala de Paula B.H., Crocamo S. (2022). Aiming for the Cure in ERBB2-Positive Metastatic Breast Cancer-Should We Go “All In”?. JAMA Oncol..

[11] Thanopoulou E., Khader L., Caira M., Wardley A., Ettl J., Miglietta F., Neven P., Guarneri V. (2020). Therapeutic Strategies for the Management of Hormone Receptor-Positive, Human Epidermal Growth Factor Receptor 2-Positive (HR+/HER2+) Breast Cancer: A Review of the Current Literature. Cancers.

[12] Gianni L., Pienkowski T., Im Y.H., Tseng L.M., Liu M.C., Lluch A., Starostawska E., Haba-Rodriguez J., Sou S.A., Pedrini J.L. (2012). Efficacy and safety of neoadjuvant pertuzumab and trastuzumab in women with locally advanced, inflammatory, or early HER2-positive breast cancer (NeoSphere): A randomised multicentre, open-label, phase 2 trial. Lancet Oncol..

[13] de Azambuja E., Holmes A.P., Piccart-Gebhart M., Holmes E., Di Cosimo S., Swaby R.F., Untvh M., Jackisch C., Lang I., Smith I. (2014). Lapatinib with trastuzumab for HER2 positive early breast cancer (NeoALTTO): Survival outcomes of a randomised, open label, multicentre phase 3 trial and their association with pathological complete response. Lancet Oncol..

[14] Schneeweiss A., Chia S., Hickish T., Harvey V., Eniu A., Hegg R., Tausch C., Seo J.H., Tsai Y.F., Ratnayake J. (2013). Pertuzumab plus trastuzumab in combination with standard neoadjuvant anthracycline-containing and anthracycline-free chemotherapy regimens in patients with HER2-positive early breast cancer: A randomized phase II cardiac safety study (TRYPHAENA). Ann. Oncol..

[15] Carey L.A., Berry D.A., Cirrincione C.T., Barry W.T., Pitcher B.N., Harris L.N., Ollila D.W., Krop I.E., Henry N.L., Weckstein D.J. (2016). Molecular heterogeneity and response to neoadjuvant human epidermal growth factor receptor 2 targeting in CALGB 40601, a randomized phase III trial of paclitaxel plus trastuzumab with or without lapatinib. J. Clin. Oncol..

[16] Untch M., Fasching P.A., Konecny G.E., Hasmüller S., Lebeau A., Kreienberg R., Camara O., Müller V., du Bois A., Kühn T. (2011). Pathologic complete response after neoadjuvant chemotherapy plus trastuzumab predicts favorable survival in human epidermal growth factor receptor 2-overexpressing breast cancer: Results from the TECHNO trial of the AGO and GBG study groups. J. Clin. Oncol..

[17] Crocamo S., Binato R., de Paula B., Vignal G., Magalhães L., Sarmento R., Accioly M.T., Small I., Gioia S., Maroun P. (2019). Ácido zoledrônico neoadjuvante para câncer de mama HER2-positivo: O estudo Zo-NAnTax. Ther. Adv. Med. Oncol..

[18] Juarez D., Fruman D.A. (2021). Targeting the Mevalonate Pathway in Cancer. Trends Cancer.

[19] Guerra B., Recio C., Aranda-Tavío H., Guerra-Rodríguez M., García-Castellano J.M., Fernández-Pérez L. (2021). The Mevalonate Pathway, a Metabolic Target in Cancer Therapy. Front. Oncol..

[20] Bathaie S.Z., Ashrafi M., Azizian M., Tamanoi F. (2017). Mevalonate Pathway and Human Cancers. Curr. Mol. Pharmacol..

[21] Wang L., Fang D., Xu J., Luo R. (2020). Various pathways of zoledronic acid against osteoclasts and bone cancer metastasis: A brief review. BMC Cancer.

[22] Rimawi M.F., Schiff R., Osborne C.K. (2015). Targeting HER2 for the Treatment of Breast Cancer. Annu Rev Med..

[23] O’Lone R., Frith M.C., Karlsson E.K., Hansen U. (2004). Genomic targets of nuclear estrogen receptors. Mol. Endocrinol..

[24] Losel R., Wehling M. (2003). Nongenomic actions of steroid hormones. Nat. Rev. Mol. Cell Biol..

[25] Vaz-Luis I., Winer E.P., Lin N.U. (2013). Human epidermal growth factor receptor-2-positive breast cancer: Does estrogen receptor status define two distinct subtypes?. Ann. Oncol..

[26] Wang Y.C., Morrison G., Gillihan R., Guo J., Ward R.M., Fu X., Botero M.f., Healy N.A., Hilsenbeck S.G., Phillips G.L. (2011). Different mechanisms for resistance to trastuzumab versus lapatinib in HER2-positive breast cancers—Role of estrogen receptor and HER2 reactivation. Breast Cancer Res..

[27] Guarneri V., Griguolo G., Miglietta F., Conte P.F., Dieci M.V., Girardi F. (2022). Survival after neoadjuvant therapy with trastuzumab-lapatinib and chemotherapy in patients with HER2-positive early breast cancer: A meta-analysis of randomized trials. ESMO Open.

[28] Schneeweiss A., Chia S., Hickish T., Harvey V., Eniu A., Waldron-Lynch M., Eng-Wong J., Kirk S., Cortés J. (2018). Long-term efficacy analysis of the randomised, phase II TRYPHAENA cardiac safety study: Evaluating pertuzumab and trastuzumab plus standard neoadjuvant anthracycline-containing and anthracycline-free chemotherapy regimens in patients with HER2-positive early breast cancer. Eur. J. Cancer.

[29] Oshi M., Takahashi H., Tokumaru Y., Yan L., Rashid O.M., Matsuyama R., Endo I., Takabe K. (2020). G2M Cell Cycle Pathway Score as a Prognostic Biomarker of Metastasis in Estrogen Receptor (ER)-Positive Breast Cancer. Int. J. Mol. Sci..

[30] Dunbier A.K., Anderson H., Ghazoui Z., Folkerd E.J., A’Hern R., Crowder R.J., Hoog J., Smith I.E., Osin P., Nerurkar A. (2010). Relationship Between Plasma Estradiol Levels and Estrogen-Responsive Gene Expression in Estrogen Receptor–Positive Breast Cancer in Postmenopausal Women. J. Clin. Oncol..

[31] Mohammed H., D’Santos C., Serandour A.A., Ali H.R., Brown G.D., Atkins A., Rueda O.M., Holmes K., Theodorou V., Robinson J.L.L. (2013). Endogenous Purification Reveals GREB1 as a Key Estrogen Receptor Regulatory Factor. Cell Rep..

[32] Haines C.N., Klingensmith H.D., Komara M., Burd C.J. (2020). GREB1 regulates PI3K/Akt signaling to control hormone-sensitive breast cancer proliferation. Carcinogenesis.

[33] Räikkönen J., Mönkkönen H., Auriola S., Mönkkönen J. (2010). Mevalonate pathway intermediates downregulate zoledronic acid-induced isopentenyl pyrophosphate and ATP analog formation in human breast cancer cells. Biochem. Pharmacol..

[34] Iizuka-Ohashi M., Watanabe M., Sukeno M., Morita M., Thi N., Hoang H.N.T.H., Kuchimaru T., Kizaka-Kondoh S., Sowa Y., Sakaguchi K. (2018). Blockage of the mevalonate pathway overcomes the apoptotic resistance to MEK inhibitors with suppressing the activation of Akt in cancer cells. Oncotarget.

[35] Attardi L.D., Reczek E.E., Cosmas C., Demicco E.G., McCurrach M.E., Lowe S.W., Jacks T. (2000). PERP, an apoptosis-associated target of p53, is a novel member of the PMP-22/gas3 family. Genes Dev..

[36] Liou G.Y., Storz P. (2010). Reactive oxygen species in cancer. Free Radic. Res..

[37] Campos L.W., Pissinato L.G., Yunes J.A. (2019). Deleterious and Oncogenic Mutations in the IL7RA. Cancers.

[38] Cardone R.A., Bellizzi A., Busco G., Weinman E.J., Dell’Aquila M.E., Casavola V., Amalia A., Mangia A., Paradiso A., Reshkin S.J. (2007). The NHERF1 PDZ2 Domain Regulates PKA–RhoA–p38-mediated NHE1 Activation and Invasion in Breast Tumor Cells. Mol. Biol Cell..

[39] Paradiso A., Scarpi E., Malfettone A., Addati T., Giotta F., Simone G., Amadori D., Mangia A. (2013). Nuclear NHERF1 expression as a prognostic marker in breast cancer. Cell Death Dis..

[40] Liu H., Ma Y., He H.W., Wang J.P., Jiang J.D., Shao R.G. (2015). SLC9A3R1 stimulates autophagy via BECN1 stabilization in breast cancer cells. Autophagy.

[41] Jeong J., Choi J., Kim W., Dann P., Takyar F., Gefter J., Friedman P.A., Wysolmerski J. (2019). Inhibition of ezrin causes PKCα-mediated internalization of erbb2/HER2 tyrosine kinase in breast cancer cells. J. Biol. Chem..

[42] Gest C., Joimel U., Huang L., Pritchard L.L., Petit A., Dulong C., Buquet C., Hu C.-Q., Miirshahi P., Larent F.M. (2013). Rac3 induces a molecular pathway triggering breast cancer cell aggressiveness: Differences in MDA-MB-231 and MCF-7 breast cancer cell lines. BMC Cancer.

[43] Chalhoub N., Baker S.J. (2009). PTEN and the PI3-Kinase Pathway in Cancer. Annu. Rev. Pathol..

[44] Rodriguez-Viciana P., Warne P.H., Vanhaesebroeck B., Waterfield M.D. (1996). Activation of phosphoinositide 3-kinase by interaction with Ras and by point mutation. EMBO J..

[45] Zhang B., Kirov S., Snoddy J. (2005). WebGestalt: An integrated system for exploring gene sets in various biological contexts. Nucleic Acids Res..

[46] Chen E.Y., Tan C.M., Kou Y., Duan Q., Wang Z., Meirelles G.V., Clark N.R., Ma’ayan A. (2013). Enrichr: Interactive and collaborative HTML5 gene list enrichment analysis tool. BMC Bioinform..

[47] Kuleshov M.V., Jones M.R., Rouillard A.D., Fernandez N.F., Duan Q., Wang Z., Koplev S., Jenkins S.L., Jagodnik K.M., Lachmann A. (2016). Enrichr: A comprehensive gene set enrichment analysis web server 2016 update. Nucleic Acids Res..

[48] Xie Z., Bailey A., Kuleshov M.V., Clarke D.J.B., Evangelista J.E., Jenkins S.L., Lachmann A., Wojciechowicz M.L., Kropiwnicki E., Jagodnik K.M. (2021). Gene Set Knowledge Discovery with Enrichr. Curr. Protoc..

[49] Szklarczyk D., Franceschini A., Wyder S., Forslund K., Heller D., Huerta-Cepas J., Simonovic M., Roth A., Alberto S., Tsafou K.P. (2015). STRING v10: Protein–protein interaction networks, integrated over the tree of life. Nucleic Acids Res..

