# Supplementary material for: Translational Results of Zo-NAnTax: A Phase II Trial of Neoadjuvant Zoledronic Acid in HER2-Positive Breast Cancer
Source: Int J Mol Sci. 2022 Dec 8;23(24):15515. doi: 10.3390/ijms232415515 (PMC9779412; doi:10.3390/ijms232415515)
Supplement: Supplementary file 1 [file ijms-23-15515-s001.zip › ijms-1994329-supplementary.pdf]

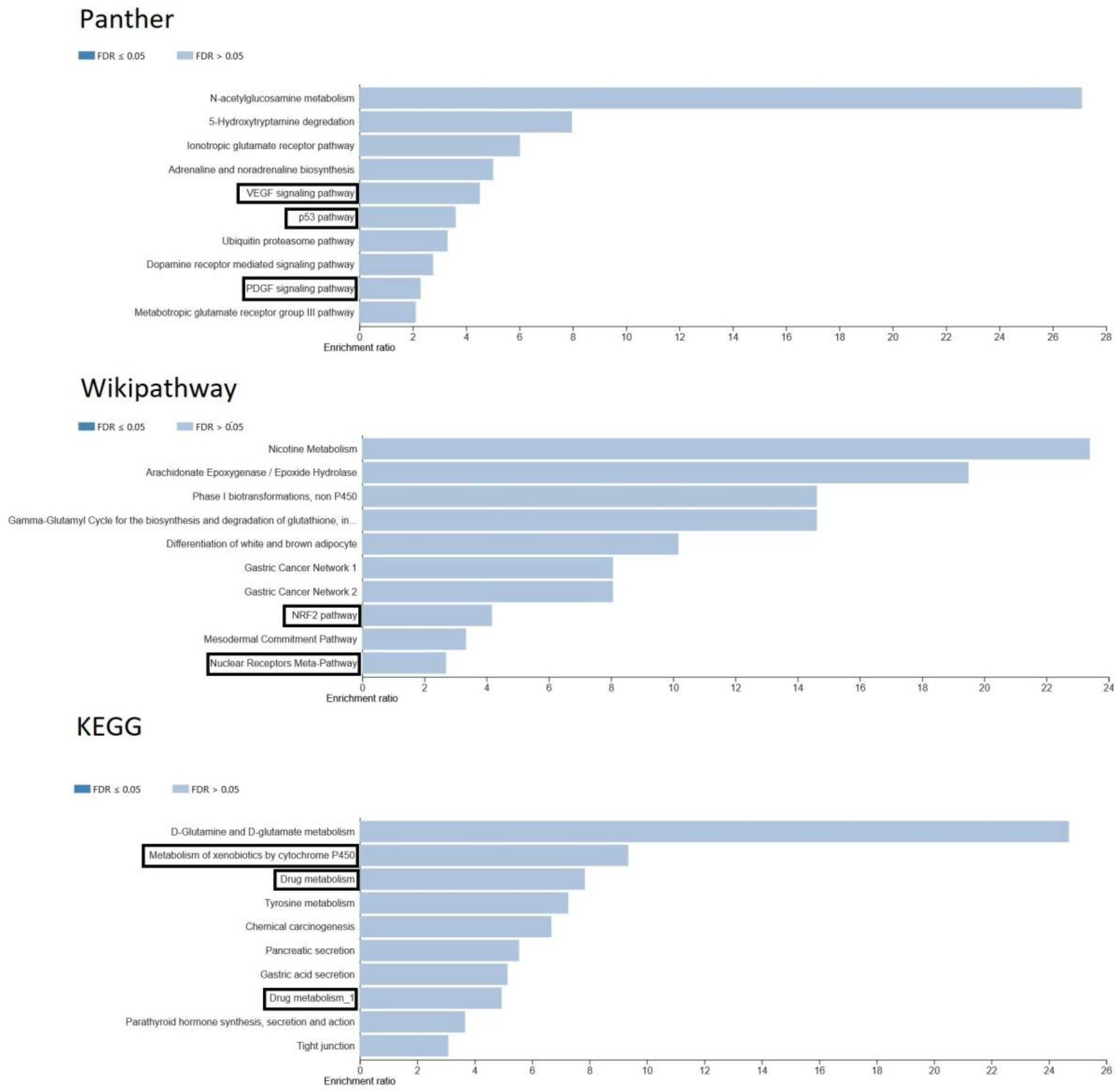

Figure S1. Top 10 signaling pathways related to the differentially expressed genes using Panther, Wikipathway, and KEGG databases. Black boxes indicate the most relevant pathways.

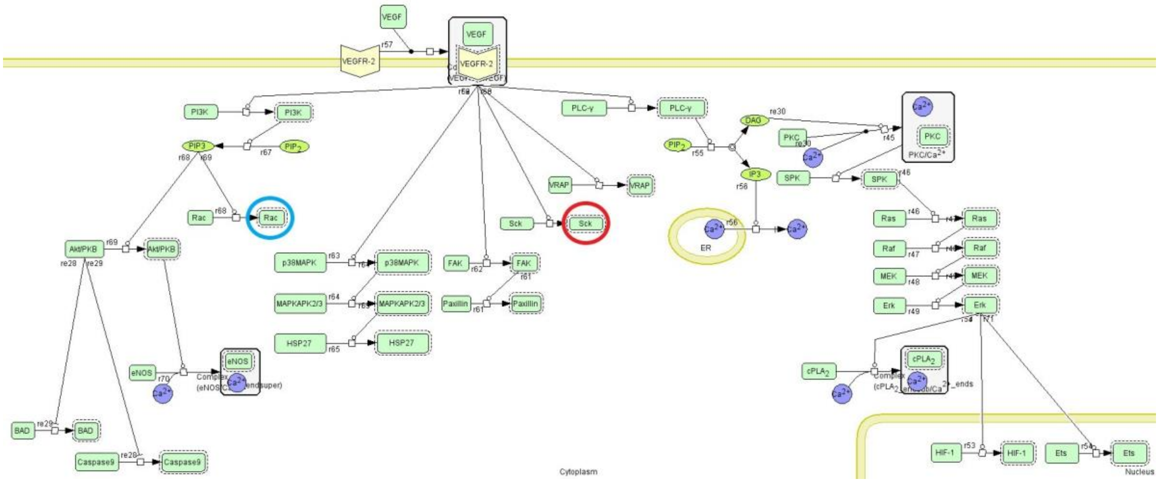

Figure S2. VEGF signaling pathway (Panther gene set: P00056). Upregulated genes are marked with red circles and downregulated genes with blue circles

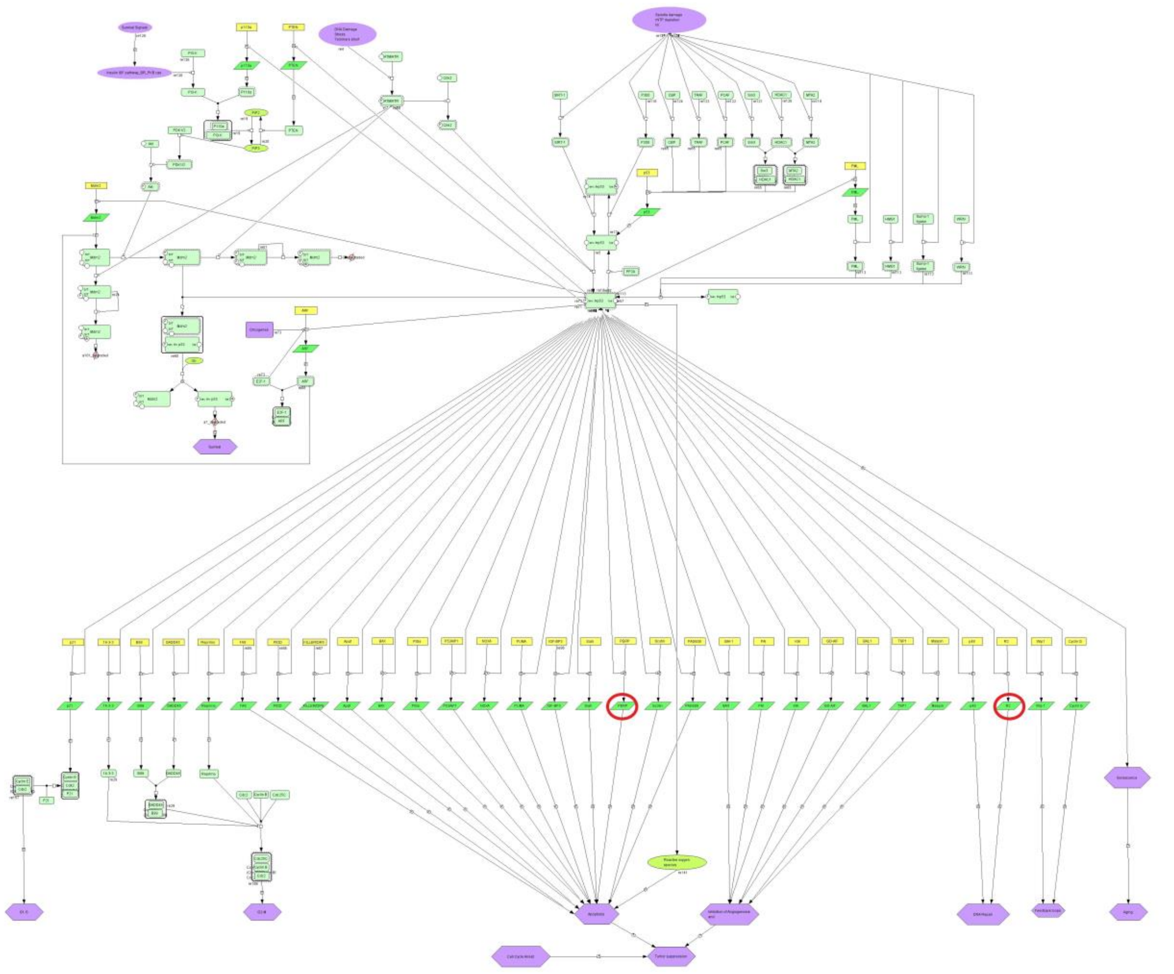

Figure S3. p53 signaling pathway (Panther gene set: P00059). Upregulated genes are marked with red circles

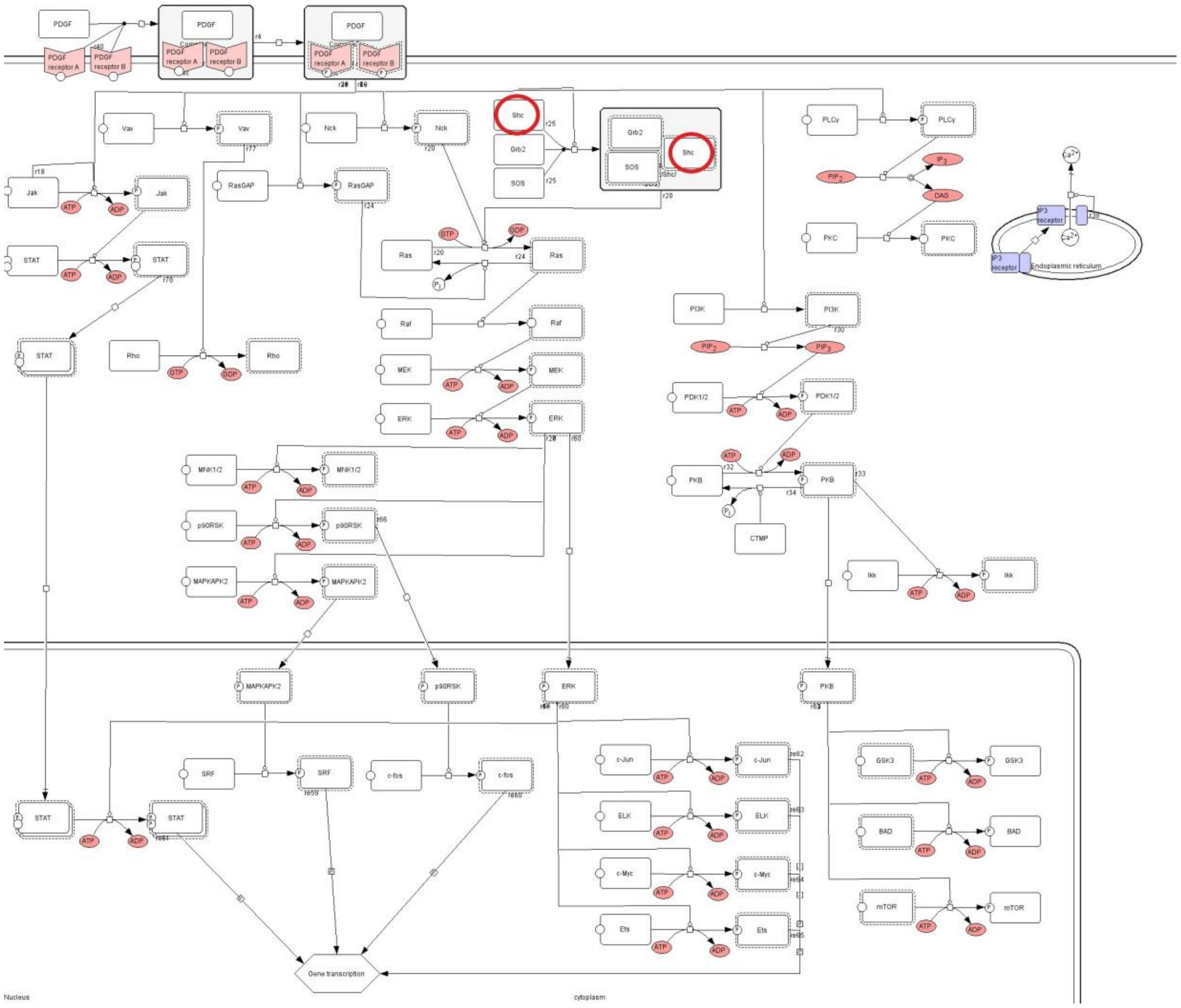

Figure S4. PDGF signaling pathway (Panther gene set: P00047). Upregulated genes are marked with red circles

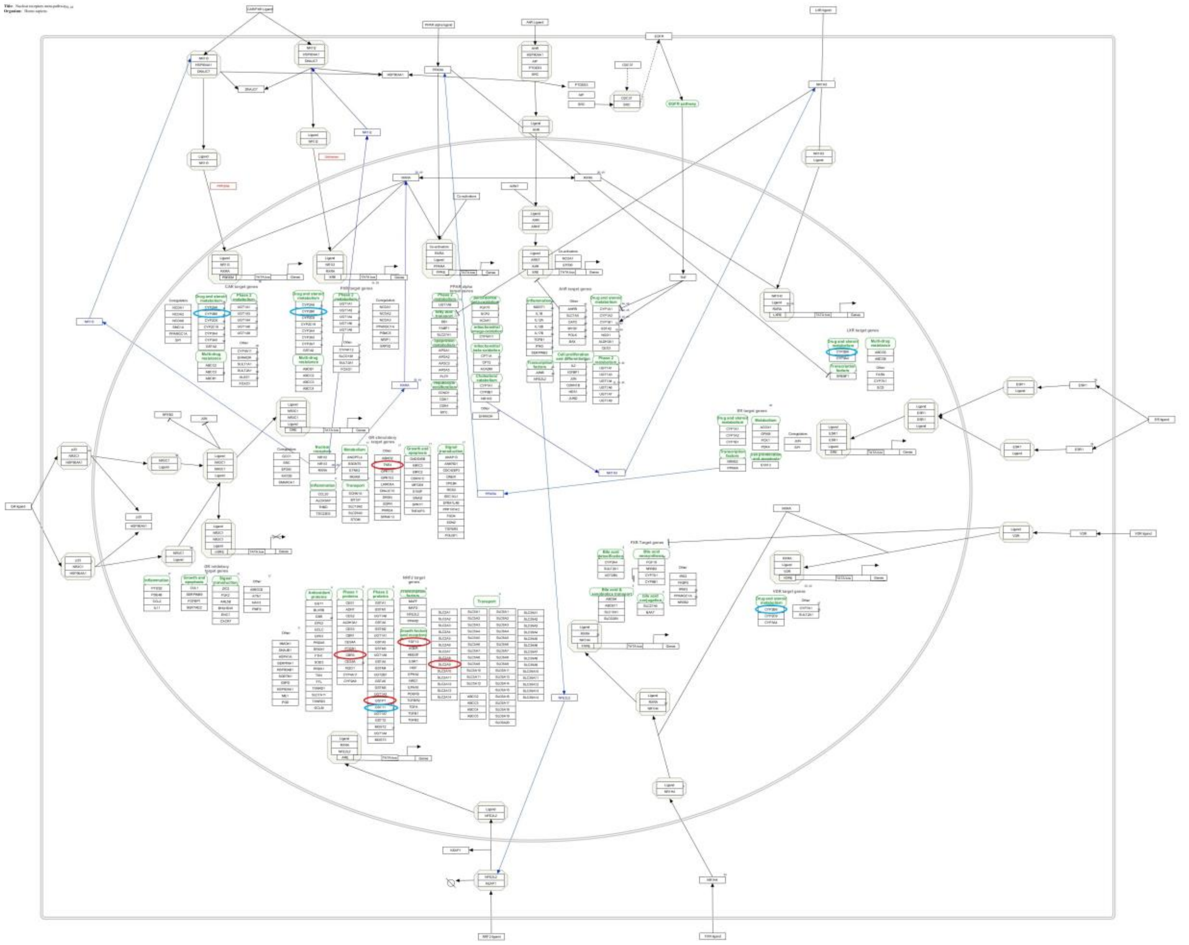

Figure S5. Nuclear receptors meta-pathway (Wikipathways gene set: WP2882). Upregulated genes are marked with red circles and downregulated genes with blue circles

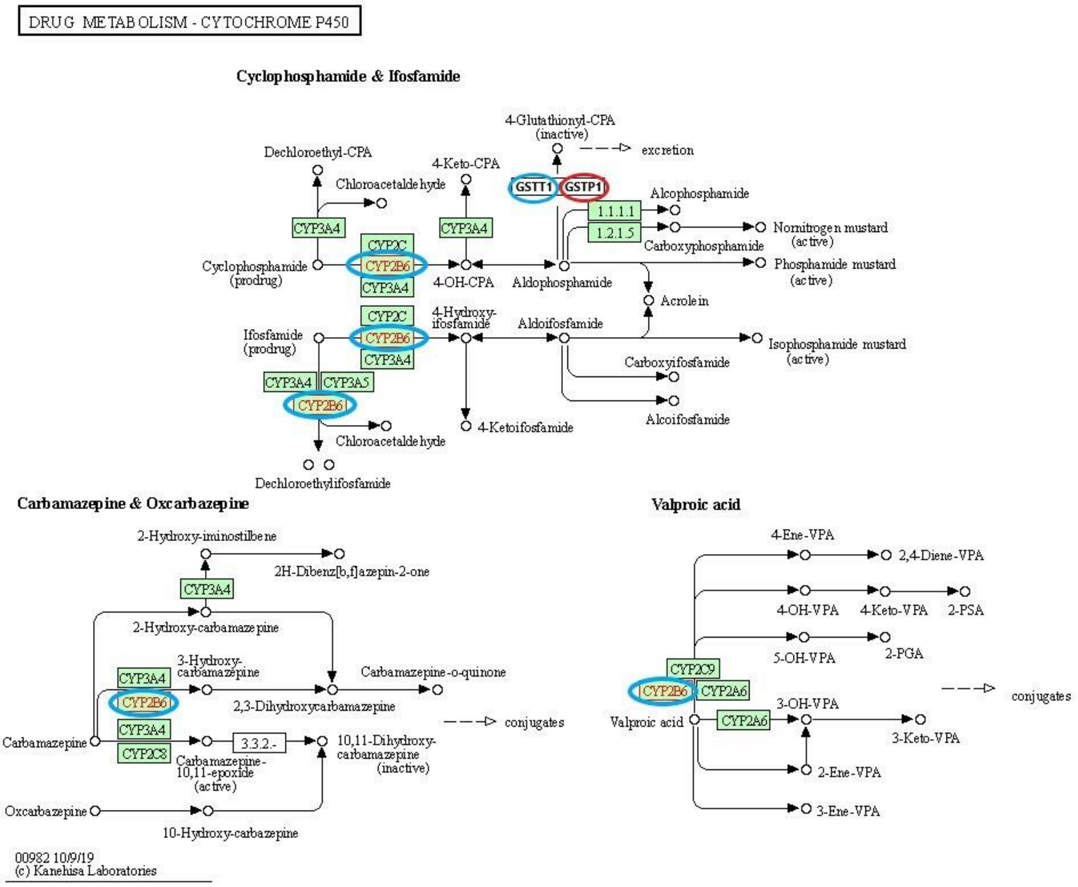

Figure S6. Drug metabolism pathway (Adapted KEGG gene set: hsa00982). Upregulated genes are marked with red circles and downregulated genes with blue circles

Table S1. - List of the 164 differentially expressed genes identified by microarray assay.

GeneSymbol.	Fold Change	Description
NKAIN1	-15,56	Homo sapiens sodium/potassium transporting ATPase interacting 1 (NKAIN1), mRNA [NM_024522]
SERPINA3	-6,88	Homo sapiens serpin family A member 3 (SERPINA3), mRNA [NM_001085]
TRIM58	-6,84	Homo sapiens tripartite motif containing 58 (TRIM58), mRNA [NM_015431]
OLFM1	-4,38	Homo sapiens olfactomedin 1 (OLFM1), transcript variant 1, mRNA [NM_014279]
CA2	-4,31	Homo sapiens carbonic anhydrase 2 (CA2), transcript variant 1, mRNA [NM_000067]
TESMIN	-4,11	Homo sapiens testis expressed metallothionein like protein (TESMIN), transcript variant 1, mRNA [NM_004923]
CYP2B6	-3,85	Homo sapiens cytochrome P450 family 2 subfamily B member 6 (CYP2B6), mRNA [NM_000767]
GATA3-AS1	-3,29	Homo sapiens GATA3 antisense RNA 1 (GATA3-AS1), transcript variant 2, long non-coding RNA [NR_024256]
PARD6B	-3,22	Homo sapiens par-6 family cell polarity regulator beta (PARD6B), mRNA [NM_032521]
PREX1	-3,19	Homo sapiens phosphatidylinositol-3,4,5-trisphosphate dependent Rac exchange factor 1 (PREX1), mRNA [NM_020820]
H3C10	-3,19	Homo sapiens histone cluster 1 H3 family member h (HIST1H3H), mRNA [NM_003536]
GREB1	-3,12	Homo sapiens growth regulating estrogen receptor binding 1 (GREB1), transcript variant a, mRNA [NM_014668]
NAT1	-3,10	Homo sapiens N-acetyltransferase 1 (NAT1), transcript variant 5, mRNA [NM_000662]
SLC16A6	-2,93	Homo sapiens solute carrier family 16 member 6 (SLC16A6), transcript variant 2, mRNA [NM_004694]
IL17RB	-2,90	Homo sapiens interleukin 17 receptor B (IL17RB), mRNA [NM_018725]
LRRCC1	-2,83	Homo sapiens leucine rich repeat and coiled-coil centrosomal protein 1 (LRRCC1), transcript variant 1, mRNA [NM_033402]
PRRT2	-2,83	Homo sapiens proline rich transmembrane protein 2 (PRRT2), transcript variant 1, mRNA [NM_145239]
MYBL1	-2,83	Homo sapiens MYB proto-oncogene like 1 (MYBL1), transcript variant 1, mRNA [NM_001080416]
TBC1D9	-2,74	Homo sapiens TBC1 domain family member 9 (TBC1D9), mRNA [NM_015130]
H3C15	-2,70	Homo sapiens histone cluster 2 H3 family member a (HIST2H3A), mRNA [NM_001005464]
GSTT1	-2,69	Homo sapiens glutathione S-transferase theta 1 (GSTT1), transcript variant 1, mRNA [NM_000853]
JPT1	-2,65	Homo sapiens Jupiter microtubule associated homolog 1 (JPT1), transcript variant 2, mRNA [NM_001002032]
P2RY2	-2,61	Homo sapiens purinergic receptor P2Y2 (P2RY2), transcript variant 1, mRNA [NM_176072]
RAC3	-2,60	Homo sapiens Rac family small GTPase 3 (RAC3), transcript variant 1, mRNA [NM_005052]
E2F7	-2,59	Homo sapiens E2F transcription factor 7 (E2F7), mRNA [NM_203394]
LRFN4	-2,56	Homo sapiens leucine rich repeat and fibronectin type III domain containing 4 (LRFN4), transcript variant 1, mRNA [NM_024036]
H4C1	-2,54	Homo sapiens histone cluster 1 H4 family member a (HIST1H4A), mRNA [NM_003538]
RSKR	-2,54	Homo sapiens ribosomal protein S6 kinase related (RSKR), mRNA [NM_001174103]
CDK5R1	-2,53	Homo sapiens cyclin dependent kinase 5 regulatory subunit 1 (CDK5R1), mRNA [NM_003885]
IFT122	-2,52	Homo sapiens intraflagellar transport 122 (IFT122), transcript variant 3, mRNA [NM_018262]
MLF1	-2,48	Homo sapiens myeloid leukemia factor 1 (MLF1), transcript variant 1, mRNA [NM_022443]
TRPS1	-2,48	Homo sapiens transcriptional repressor GATA binding 1 (TRPS1), transcript variant 1, mRNA [NM_014112]
ATAD5	-2,42	Homo sapiens ATPase family AAA domain containing 5 (ATAD5), mRNA [NM_024857]
SLC9A3R1	-2,42	Homo sapiens SLC9A3 regulator 1 (SLC9A3R1), mRNA [NM_004252]
ZC4H2	-2,40	Homo sapiens zinc finger C4H2-type containing (ZC4H2), transcript variant 1, mRNA [NM_018684]

RDM1	-2,39	Homo sapiens RAD52 motif containing 1 (RDM1), transcript variant 2, mRNA [NM_001034836]
KIF5C	-2,37	Homo sapiens kinesin family member 5C (KIF5C), transcript variant 1, mRNA [NM_004522]
H3C2	-2,37	Homo sapiens histone cluster 1 H3 family member b (HIST1H3B), mRNA [NM_003537]
SLC1A2	-2,36	Homo sapiens solute carrier family 1 member 2 (SLC1A2), transcript variant 1, mRNA [NM_004171]
UBE2C	-2,35	Homo sapiens ubiquitin conjugating enzyme E2 C (UBE2C), transcript variant 1, mRNA [NM_007019]
FAM155A	-2,33	family with sequence similarity 155 member A [Source:HGNC Symbol;Acc:HGNC:33877] [ENST00000375915]
C17orf58	-2,33	Homo sapiens chromosome 17 open reading frame 58 (C17orf58), transcript variant 1, mRNA [NM_181655]
ZNF689	-2,31	Homo sapiens zinc finger protein 689 (ZNF689), transcript variant 3, non-coding RNA [NR_073481]
SPAG5	-2,28	Homo sapiens sperm associated antigen 5 (SPAG5), mRNA [NM_006461]
RERG	-2,28	Homo sapiens RAS like estrogen regulated growth inhibitor (RERG), transcript variant 1, mRNA [NM_032918]
MBOAT1	-2,27	Homo sapiens membrane bound O-acyltransferase domain containing 1 (MBOAT1), transcript variant 2, non-coding RNA [NR_073465]
GGH	-2,25	Homo sapiens gamma-glutamyl hydrolase (GGH), mRNA [NM_003878]
PAAF1	-2,22	Homo sapiens proteasomal ATPase associated factor 1 (PAAF1), transcript variant 6, mRNA [NM_001363556]
GATA3	-2,21	Homo sapiens GATA binding protein 3 (GATA3), transcript variant 1, mRNA [NM_001002295]
AMZ2P1	-2,20	Homo sapiens archaelysin family metallopeptidase 2 pseudogene 1 (AMZ2P1), non-coding RNA [NR_026903]
EMB	-2,17	Homo sapiens embigin (EMB), mRNA [NM_198449]
LMNTD2-AS1	-2,15	Homo sapiens uncharacterized LOC692247 (LOC692247), transcript variant 1, long non-coding RNA [NR_147607]
GNA13	-2,15	Homo sapiens G protein subunit alpha 13 (GNA13), transcript variant 1, mRNA [NM_006572]
SERPINA11	-2,13	Homo sapiens serpin family A member 11 (SERPINA11), mRNA [NM_001080451]
CCDC106	-2,13	Homo sapiens coiled-coil domain containing 106 (CCDC106), transcript variant 5, mRNA [NM_001370470]
LINC02076	-2,12	Homo sapiens long intergenic non-protein coding RNA 2076 (LINC02076), long non-coding RNA [NR_104343]
NUMBL	-2,11	Homo sapiens NUMB like endocytic adaptor protein (NUMBL), transcript variant 1, mRNA [NM_004756]
RNF213	-2,10	Homo sapiens ring finger protein 213 (RNF213), transcript variant 2, mRNA [NM_020954]
KLHDC9	-2,09	Homo sapiens kelch domain containing 9 (KLHDC9), transcript variant 2, mRNA [NM_001007255]
SCARNA16	-2,08	Homo sapiens small Cajal body-specific RNA 16 (SCARNA16), guide RNA [NR_003013]
FAM122B	-2,08	Homo sapiens family with sequence similarity 122B (FAM122B), transcript variant 6, mRNA [NM_001331088]
ZNF703	-2,08	Homo sapiens zinc finger protein 703 (ZNF703), mRNA [NM_025069]
COA4	-2,07	Homo sapiens cytochrome c oxidase assembly factor 4 homolog (COA4), mRNA [NM_016565]
LOC644656	-2,06	Homo sapiens uncharacterized LOC644656 (LOC644656), long non-coding RNA [NR_036539]
STAU2	-2,06	Homo sapiens staufen double-stranded RNA binding protein 2 (STAU2), transcript variant 1, mRNA [NM_001164380]
RPL22L1	-2,06	Homo sapiens ribosomal protein L22 like 1 (RPL22L1), transcript variant 1, mRNA [NM_001099645]
NLK	-2,06	Homo sapiens nemo like kinase (NLK), mRNA [NM_016231]

ATAD2	-2,06	Homo sapiens ATPase family AAA domain containing 2 (ATAD2), mRNA [NM_014109]
CMSS1	-2,06	Homo sapiens cms1 ribosomal small subunit homolog (CMSS1), transcript variant 1, mRNA [NM_032359]
ABCA3	-2,06	Homo sapiens ATP binding cassette subfamily A member 3 (ABCA3), mRNA [NM_001089]
EIF4A3	-2,05	Homo sapiens eukaryotic translation initiation factor 4A3 (EIF4A3), mRNA [NM_014740]
NPAP1	-2,05	Homo sapiens nuclear pore associated protein 1 (NPAP1), mRNA [NM_018958]
FAM72B	-2,05	Homo sapiens family with sequence similarity 72 member B (FAM72B), transcript variant 1, mRNA [NM_001100910]
ZNF814	-2,04	Homo sapiens zinc finger protein 814 (ZNF814), mRNA [NM_001144989]
C3orf14	-2,04	Homo sapiens chromosome 3 open reading frame 14 (C3orf14), transcript variant 1, mRNA [NM_020685]
LINC0046 7	-2,01	Homo sapiens long intergenic non-protein coding RNA 467 (LINC00467), long non-coding RNA [NR_026761]
ZHX1-C8orf76	-2,01	Homo sapiens ZHX1-C8orf76 readthrough (ZHX1-C8orf76), mRNA [NM_001204180]
NDUFAF8	-2,00	Homo sapiens NADH:ubiquinone oxidoreductase complex assembly factor 8 (NDUFAF8), transcript variant 3, mRNA [NM_001353403]
LPIN1	2,00	Homo sapiens lipin 1 (LPIN1), transcript variant 1, mRNA [NM_145693]
FGF13	2,01	Homo sapiens fibroblast growth factor 13 (FGF13), transcript variant 3, mRNA [NM_001139501]
ANO7	2,02	Homo sapiens anoctamin 7 (ANO7), transcript variant NGEP-L, mRNA [NM_001001891]
TMEM123	2,02	Homo sapiens transmembrane protein 123 (TMEM123), mRNA [NM_052932]
YBEY	2,03	Homo sapiens ybeY metalloendoribonuclease (YBEY), transcript variant 6, mRNA [NM_001314025]
SLC2A9	2,03	Homo sapiens solute carrier family 2 member 9 (SLC2A9), transcript variant 2, mRNA [NM_001001290]
TSPAN11	2,05	Homo sapiens tetraspanin 11 (TSPAN11), transcript variant 3, mRNA [NM_001370302]
GFPT1	2,05	Homo sapiens glutamine--fructose-6-phosphate transaminase 1 (GFPT1), transcript variant 1, mRNA [NM_001244710]
ADGRG1	2,06	Homo sapiens adhesion G protein-coupled receptor G1 (ADGRG1), transcript variant 3, mRNA [NM_201525]
SHC2	2,07	Homo sapiens SHC adaptor protein 2 (SHC2), mRNA [NM_012435]
RASSF4	2,07	Homo sapiens Ras association domain family member 4 (RASSF4), mRNA [NM_032023]
COMT	2,07	Homo sapiens catechol-O-methyltransferase (COMT), transcript variant 1, mRNA [NM_000754]
TRAF3IP3	2,10	Homo sapiens TRAF3 interacting protein 3 (TRAF3IP3), transcript variant 1, mRNA [NM_025228]
ANKS6	2,11	Homo sapiens ankyrin repeat and sterile alpha motif domain containing 6 (ANKS6), mRNA [NM_173551]
CAMK2D	2,12	Homo sapiens calcium/calmodulin dependent protein kinase II delta (CAMK2D), transcript variant 3, mRNA [NM_001221]
KIF13B	2,13	Homo sapiens kinesin family member 13B (KIF13B), mRNA [NM_015254]
ARHGAP1 0	2,13	Rho GTPase activating protein 10 [Source:HGNC Symbol;Acc:HGNC:26099] [ENST00000507661]
GPCPD1	2,13	glycerophosphocholine phosphodiesterase 1 [Source:HGNC Symbol;Acc:HGNC:26957] [ENST00000379019]
SMPDL3A	2,15	Homo sapiens sphingomyelin phosphodiesterase acid like 3A (SMPDL3A), transcript variant 1, mRNA [NM_006714]
BMP7	2,17	Homo sapiens bone morphogenetic protein 7 (BMP7), mRNA [NM_001719]
PERP	2,20	Homo sapiens p53 apoptosis effector related to PMP22 (PERP), mRNA [NM_022121]
SIRPA	2,20	Homo sapiens signal regulatory protein alpha (SIRPA), transcript variant 1, mRNA [NM_001040022]
RIPK4	2,22	Homo sapiens receptor interacting serine/threonine kinase 4 (RIPK4), mRNA [NM_020639]
DGLUCY	2,26	Homo sapiens D-glutamate cyclase (DGLUCY), transcript variant 3, mRNA [NM_024952]

KIF1B	2,26	Homo sapiens kinesin family member 1B (KIF1B), transcript variant 2, mRNA [NM_183416]
CBR3	2,26	Homo sapiens carbonyl reductase 3 (CBR3), mRNA [NM_001236]
PTPRN2	2,27	Homo sapiens protein tyrosine phosphatase receptor type N2 (PTPRN2), transcript variant 1, mRNA [NM_002847]
ARL4A	2,28	Homo sapiens ADP ribosylation factor like GTPase 4A (ARL4A), transcript variant 1, mRNA [NM_005738]
ADGRE5	2,28	Homo sapiens adhesion G protein-coupled receptor E5 (ADGRE5), transcript variant 1, mRNA [NM_078481]
SRPX	2,28	Homo sapiens sushi repeat containing protein X-linked (SRPX), transcript variant 1, mRNA [NM_006307]
KCNQ1	2,29	Homo sapiens potassium voltage-gated channel subfamily Q member 1 (KCNQ1), transcript variant 1, mRNA [NM_000218]
SLC4A11	2,30	Homo sapiens solute carrier family 4 member 11 (SLC4A11), transcript variant 2, mRNA [NM_032034]
SPNS2	2,30	Homo sapiens sphingolipid transporter 2 (SPNS2), mRNA [NM_001124758]
ADI1	2,30	Homo sapiens acireductone dioxygenase 1 (ADI1), transcript variant 1, mRNA [NM_018269]
FAM20A	2,31	Homo sapiens FAM20A golgi associated secretory pathway pseudokinase (FAM20A), transcript variant 1, mRNA [NM_017565]
TNFRSF25	2,32	Homo sapiens TNF receptor superfamily member 25 (TNFRSF25), transcript variant 1, mRNA [NM_148965]
PM20D2	2,34	Homo sapiens peptidase M20 domain containing 2 (PM20D2), mRNA [NM_001010853]
RNF145	2,34	Homo sapiens ring finger protein 145 (RNF145), transcript variant 2, mRNA [NM_144726]
IL7R	2,34	Homo sapiens interleukin 7 receptor (IL7R), transcript variant 1, mRNA [NM_002185]
METTL7A	2,35	Homo sapiens methyltransferase like 7A (METTL7A), mRNA [NM_014033]
PPARGC1B	2,35	Homo sapiens PPARG coactivator 1 beta (PPARGC1B), transcript variant 1, mRNA [NM_133263]
MAP1B	2,36	Homo sapiens microtubule associated protein 1B (MAP1B), transcript variant 1, mRNA [NM_005909]
SH3BGRL	2,39	Homo sapiens SH3 domain binding glutamate rich protein like (SH3BGRL), mRNA [NM_003022]
GRHL3	2,42	Homo sapiens grainyhead like transcription factor 3 (GRHL3), transcript variant 2, mRNA [NM_198173]
CD82	2,43	Homo sapiens CD82 molecule (CD82), transcript variant 1, mRNA [NM_002231]
TACC2	2,43	Homo sapiens transforming acidic coiled-coil containing protein 2 (TACC2), transcript variant 1, mRNA [NM_206862]
MANBA	2,43	mannosidase beta [Source:HGNC Symbol;Acc:HGNC:6831] [ENST00000505239]
MPHOSPH6	2,43	Homo sapiens M-phase phosphoprotein 6 (MPHOSPH6), mRNA [NM_005792]
GGTLC1	2,44	Homo sapiens gamma-glutamyltransferase light chain 1 (GGTLC1), transcript variant B, mRNA [NM_178312]
AIF1L	2,46	Homo sapiens allograft inflammatory factor 1 like (AIF1L), transcript variant 1, mRNA [NM_031426]
GPM6B	2,47	Homo sapiens glycoprotein M6B (GPM6B), transcript variant 1, mRNA [NM_001001995]
HOXA3	2,48	Homo sapiens homeobox A3 (HOXA3), transcript variant 2, mRNA [NM_153631]
TACSTD2	2,48	tumor associated calcium signal transducer 2 [Source:HGNC Symbol;Acc:HGNC:11530] [ENST00000371225]
C2orf72	2,52	Homo sapiens chromosome 2 open reading frame 72 (C2orf72), mRNA [NM_001144994]
KCNN4	2,53	Homo sapiens potassium calcium-activated channel subfamily N member 4 (KCNN4), mRNA [NM_002250]
LOC388242	2,53	Homo sapiens SAGA complex associated factor 29 pseudogene (LOC388242), non-coding RNA [NR_002556]

CDKN1C	2,57	Homo sapiens cyclin dependent kinase inhibitor 1C (CDKN1C), transcript variant 1, mRNA [NM_000076]
LRIG3	2,59	Homo sapiens leucine rich repeats and immunoglobulin like domains 3 (LRIG3), transcript variant 2, mRNA [NM_153377]
VASN	2,70	Homo sapiens vasorin (VASN), mRNA [NM_138440]
BEX4	2,71	Homo sapiens brain expressed X-linked 4 (BEX4), transcript variant 1, mRNA [NM_001127688]
MFSD2A	2,73	Homo sapiens major facilitator superfamily domain containing 2A (MFSD2A), transcript variant 8, mRNA [NM_001349823]
ALDH1A3	2,78	Homo sapiens aldehyde dehydrogenase 1 family member A3 (ALDH1A3), transcript variant 1, mRNA [NM_000693]
IRAK2	2,78	Homo sapiens interleukin 1 receptor associated kinase 2 (IRAK2), mRNA [NM_001570]
ATP2A3	2,78	Homo sapiens ATPase sarcoplasmic/endoplasmic reticulum Ca2+ transporting 3 (ATP2A3), transcript variant 5, mRNA [NM_174953]
PON3	2,82	Homo sapiens paraoxonase 3 (PON3), mRNA [NM_000940]
LONRF3	2,99	PREDICTED: Homo sapiens LON peptidase N-terminal domain and ring finger 3 (LONRF3), transcript variant X6, misc_RNA [XR_001755725]
TUBA4A	3,02	Homo sapiens tubulin alpha 4a (TUBA4A), transcript variant 1, mRNA [NM_006000]
CGNL1	3,07	Homo sapiens cingulin like 1 (CGNL1), transcript variant 2, mRNA [NM_032866]
ZNF503	3,08	Homo sapiens zinc finger protein 503 (ZNF503), transcript variant 1, mRNA [NM_032772]
LGALS3	3,09	Homo sapiens galectin 3 (LGALS3), transcript variant 1, mRNA [NM_002306]
ZNF503-AS2	3,11	Homo sapiens ZNF503 antisense RNA 2 (ZNF503-AS2), transcript variant 5, long non-coding RNA [NR_110301]
CD5	3,15	Homo sapiens CD5 molecule (CD5), transcript variant 1, mRNA [NM_014207]
SLC12A2	3,19	Homo sapiens solute carrier family 12 member 2 (SLC12A2), transcript variant 1, mRNA [NM_001046]
FAM13A	3,23	Homo sapiens family with sequence similarity 13 member A (FAM13A), transcript variant 1, mRNA [NM_014883]
LIMCH1	3,25	Homo sapiens LIM and calponin homology domains 1 (LIMCH1), transcript variant 1, mRNA [NM_014988]
OGDHL	3,32	Homo sapiens oxoglutarate dehydrogenase like (OGDHL), transcript variant 1, mRNA [NM_018245]
TSPAN8	3,34	Homo sapiens tetraspanin 8 (TSPAN8), transcript variant 1, mRNA [NM_004616]
UCHL1	3,49	ubiquitin C-terminal hydrolase L1 [Source:HGNC Symbol;Acc:HGNC:12513] [ENST00000472501]
TNS4	3,54	Homo sapiens tensin 4 (TNS4), mRNA [NM_032865]
ST6GALNAC1	3,71	Homo sapiens ST6 N-acetylgalactosaminide alpha-2,6-sialyltransferase 1 (ST6GALNAC1), transcript variant 1, mRNA [NM_018414]
SERHL2	3,73	Homo sapiens serine hydrolase like 2 (SERHL2), transcript variant 1, mRNA [NM_014509]
KRBOX1	3,79	Homo sapiens KRAB box domain containing 1 (KRBOX1), mRNA [NM_001205272]
SLPI	3,81	Homo sapiens secretory leukocyte peptidase inhibitor (SLPI), mRNA [NM_003064]
GSTP1	4,02	Homo sapiens glutathione S-transferase pi 1 (GSTP1), mRNA [NM_000852]
PSCA	5,49	Homo sapiens prostate stem cell antigen (PSCA), transcript variant 1, mRNA [NM_005672]
MUCL1	5,56	Homo sapiens mucin like 1 (MUCL1), mRNA [NM_058173]
